# Life After COVID-19: Rethinking the Healthcare System and Valuing the Role of Citizens' Engagement in Health Prevention

**DOI:** 10.3389/fpsyg.2020.589249

**Published:** 2020-10-29

**Authors:** Floriana D'Ambrosio, Antonio Giulio de Belvis, Alisha Morsella, Greta Castellini, Guendalina Graffigna, Patrizia Laurenti

**Affiliations:** ^1^Section of Hygiene, University Department of Health Sciences and Public Health, Università Cattolica del Sacro Cuore, Rome, Italy; ^2^Critical Pathways and Outcomes Evaluation Unit, Fondazione Policlinico Universitario A. Gemelli IRCCS, Rome, Italy; ^3^Department of Psychology, EngageMinds HUB – Consumer, Food & Health Engagement Research Center, Università Cattolica del Sacro Cuore, Milan, Italy; ^4^Department of Woman and Child Health and Public Health - Public Health Area, Fondazione Policlinico Universitario A. Gemelli IRCCS, Rome, Italy

**Keywords:** community-centered care, patient health engagement, healthcare engagement, citizen engagement, healthcare system, COVID-19

On December 2019, the city of Wuhan, Hubei Province in China, was hit by an unexplainably aggressive pneumonia with unknown origin (Lu et al., 2020).

Its initially rapid spread was then imputed to a novel class of coronavirus, Sars-CoV-2 and, on February 11th 2020, the disease was named Covid-19. In the following months, the viral transmission increased exponentially to the entire country and all around the world and the outbreak was officially declared a pandemic by the World Health Organization (WHO) on 11th March (Lu et al., 2020; Sohrabi et al., 2020).

As it shows, the first month of the new decade have been dominated by an unprecedented emergency which has called for a timely and exhausting response on behalf of governments and policy-makers stressing, now more than ever, the impellent need to strengthen the capacity of national healthcare systems. While the world watched all social, economic, and productive sectors drastically decelerating their pace, Intensive Care Units, diagnostic laboratories, General Practitioner surgeries, and all other healthcare services found themselves over-pressurized and working their fingers to the bone, entailing an over-exposition to burn-out and psychosocial risks of the workforce. According to the Health System Response Monitor platform by the European Observatory (European Observatory on Health Care Systems and Policies, 2020), many European national governments have been mobilizing special funds to increase workforce capacity or pay overtime to their healthcare workers. Some countries have responded to the sudden change in demand by timely reorganizing hospitals through shifts in resource allocation and, in some cases, private funding and donations have played a significant role in increasing ICU capacity. Other sectors have been also called to action via solicitations of industrial reconversion to respond to the shortage of Personal Protective Equipment (PPE), in a desperate attempt to safeguard hospitals where conditions are, in some cases, so desperate that patients are laid on floor mattresses (Nacoti et al., 2020). Of course, to save healthcare facilities from being the main vectors of Covid-19 spreads, as suspected to be the case for some hospitals in Italy, the use of appropriate PPE and sanitization procedures must occur alongside correct preventive behavior in the workplace. Integrating such comportments into routine clinical practice is the result of proactive behavior on behalf of physicians, nurses, and all other healthcare professionals however, ensuring that healthcare facilities remain safe environments is a responsibility also of patients and their caregivers. This also requires a shift in patients' attitudes and approaches toward their healthcare management, in the direction of better engagement.

The pandemic stressed the importance of rethinking and rescheduling our entire healthcare system. Western health care systems have been built around the concept of patient-centered care, but an epidemic requires a change of perspective toward a concept of community-centered care (Nacoti et al., 2020). To improve the quality of health services and their abilities to face new future challenges we need to focus on the importance of community, that must be at the center of activities of the health care system. This infection proved that diseases affect not only a single patient but also entire communities, putting a strain on our entire system if not prepared. It is therefore important to invest in public health (Heymann and Shindo, 2020), with the coordination of several complementary professional figures, such as social scientists, epidemiologists, experts in logistics, psychologists, and social workers, to be better prepared not only in response of future pandemic like this, but also in the daily adoption of correct and effective behaviors to prevent the onset of new infections. Furthermore, the role of each singular citizen in preventing the risk of contagion for him/herself and his/her community is underlined: in the absence of a vaccination against the virus contagion, the main preventive strategies are behavioral and require a high adherence to the preventive norms such as physical distancing, wearing masks and hands hygiene (Bombard et al., 2018). The adherence of citizens to such requirements require a deep shift in their approach to health and health behaviors in the direction of an enhanced engagement in one own self-management. We have a proof of this phenomenon from a survey conducted on a representative sample of 1,000 Italians in the first week of the Covid19 emergency in Italy (Graffigna et al., 2020a,b), aimed at exploring the role of patient's psychological predisposition to engage in prevention (Patient Health Engagement—PHE) (Graffigna et al., 2017) in dealing with Covid-19 emergency. PHE has previously been found to have a protective role for better clinical, psychological, and organizational outcomes (Graffigna et al., 2015). In this model, people pass from being completely overwhelmed by the crisis situation (Position of Arousal), to regain a proactive role in taking the reins of their health and positively manage it (Position of Eudemonic Project). We found that citizens with higher levels of PHE score significantly differ in terms of their trust in the healthcare system, in the healthcare professional ability to face the pandemic, in the Government measures to face the pandemic, on scientific research and on their own role and ability for preventing the diffusion of the virus. Furthermore, patients with higher levels of PHE value more the option of vaccination (Table 1) (Graffigna et al., 2020a,b).

**Table 1:** The percentage of responses for patient health engagement levels.

		**Engagement level**		
**Items**	**Total sample (*n* = 1,000)**	**Arousal (*n* = 230)**	**Adhesion (*n* = 612)**	**Eudaimonic (*n* = 158)**
*I am the main responsible for preventing the risk of contagion from new Coronavirus (COVID-19)*				
Completely agree	21%	17%	18%^*^	35%^***^
*I think vaccines are effective in preventing/treating the diseases for which they were developed*.				
Completely agree	33%	28%	32%	42%^**^
*I have full trust in scientific research*				
Completely agree	35%	28%^**^	34%	51%^***^
*I have full trust in healthcare system*				
Completely agree	17%	11%^**^	16%	31%^***^
*I have full trust in institutions*				
Completely agree	7%	4%^*^	6%	13%^***^
*The government and authorities are effectively managing the spread of the new Coronavirus (COVID-19) in Italy*				
Completely agree	9%	6%	9%	15%^***^
*The National Health System is acting in the best way to contain the spread of the new Coronavirus infection (COVID-19)*				
Completely agree	19%	11%^***^	19%	29%^***^

(1) Only the percentages of those who responded “completely agree” (5 point on a likert scale) were reported; (2) Significance in marked with asterisks (

a *significance at p < 0.05;

** significance at p < 0.01;

*** *significance at p < 0.001). Data are an extract from a broader research reported in Graffigna et al. (2020a,b)*.

In this perspective, it is fundamental that physicians, patients and caregivers become aware of the front-line role that they play in the containment of Covid-19 and are engaged in unison in the adoption of epidemiologically correct behaviors, until such actions are consolidated once and for all into organizational culture.

Working on improving the engagement is an opportunity to better orient not only the management of the actual emergency of COVID-19 but also its results on the habits and consumption of health care.

Improving the level of engagement of the health care workers may help to increase the ability to respond to all daily challenge and adherence to preventive measures, also in relation to Health Care Associated Infections (HCAIs) that the pandemic has not erased and that will come to the attention with all their lethality load.

## Author Contributions

All Authors have contributed substantially to this opinion article and have approved the final version of the manuscript.

## Conflict of Interest

The authors declare that the research was conducted in the absence of any commercial or financial relationships that could be construed as a potential conflict of interest.
